# Evaluation of foot posture development in children between three and eleven years of age using the foot posture index

**DOI:** 10.1186/1757-1146-3-S1-P17

**Published:** 2010-12-20

**Authors:** Rosemary Targett, Ian Mathieson

**Affiliations:** 1 University of Wales Institute, Cardiff, Cardiff, UK

## 

Children are commonly brought into clinic by parents anxious over ‘flat feet’. Whilst flat feet may represent a normal developmental process, the natural history of foot posture in children is incompletely understood. This fuels controversy concerning which foot postures necessitate clinical intervention. The persistence of this controversy may be due, at least in part, to the absence of a clinically convenient, reliable and valid, foot posture classification system that can be used to investigate development in large groups of children. The *Foot Posture Index* has helped alleviate many of these concerns, and was used in this study to investigate normal foot development in a group of 225 children aged between 3 and 11, selected on the basis of their attendance at a single UK primary school. Results suggested a tendency for the pronated foot posture common in 3 year olds to decrease up to eleven years of age suggesting a gradual resolution of the pronated posture associated with flat feet. However, this trend was not linear across the age groups assessed, with a range of foot postures were seen in each group. By focusing on 3 to 11 year olds and not including older children in the study it was not possible to investigate the suggestion from the data – in the form of a larger change in foot posture in 10 to 11 year olds – that important changes occur around this age. Further research is required to provide more comprehensive information to inform clinical decision making.

